# Effect of the Ion, Solvent, and Thermal Interaction Coefficients on Battery Voltage

**DOI:** 10.1021/jacs.3c11589

**Published:** 2024-02-10

**Authors:** Øystein Gullbrekken, Astrid Fagertun Gunnarshaug, Anders Lervik, Signe Kjelstrup, Sondre Kvalvåg Schnell

**Affiliations:** † Department of Materials Science and Engineering, Norwegian University of Science and Technology, NTNU, N-7491 Trondheim, Norway; ‡ PoreLab, Department of Chemistry, Norwegian University of Science and Technology, NTNU, N-7491 Trondheim, Norway

## Abstract

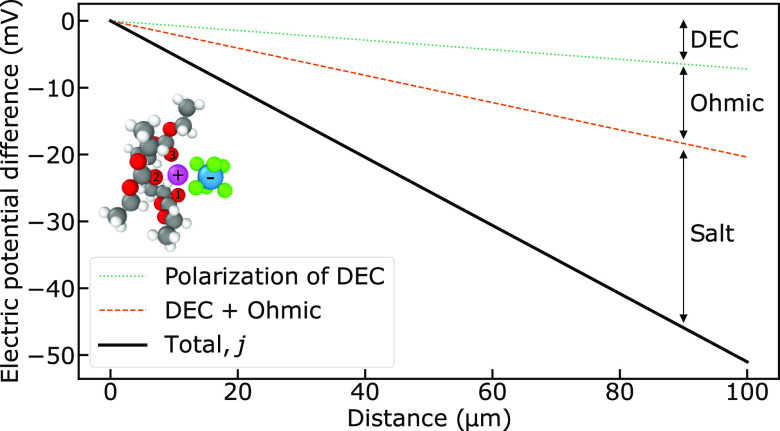

In order to increase the adoption of batteries for sustainable transport and energy storage, improved charging and discharging capabilities of lithium-ion batteries are necessary. To achieve this, accurate data that describe the internal state of the cells are essential. Several models have been derived, and transport coefficients have been reported for use in these models. We report for the first time a complete set of transport coefficients to model the concentration and temperature polarization in a lithium-ion battery ternary electrolyte, allowing us to test common assumptions. We include effects due to gradients in chemical potentials and in temperature. We find that the voltage contributions due to salt and solvent polarization are of the same order of magnitude as the ohmic loss and must be taken into account for more accurate modeling and understanding of battery performance. We report new Soret and Seebeck coefficients and find thermal polarization to be significant in cases relevant to battery research. The analysis is suitable for electrochemical systems, in general.

## Introduction

It is generally known that charging or discharging of batteries may lead to concentration polarization, i.e., changes in electrolyte composition due to an electric field.^1^ Thermal polarization, i.e., composition changes due to temperature gradients, may also play a role. The magnitudes of both follow from the transport of charge, mass, and heat in the electrolyte, including the coupling effects of these processes. The values of the coupling coefficients are central for the prediction of thermal and concentration polarization according to nonequilibrium thermodynamics, the method chosen for the present analysis.

A major part of the battery voltage is determined by the difference in electrode potentials between the cathode and the anode. In addition, ohmic resistance and polarization of the electrolyte contribute to the total cell voltage. At high charge and discharge rates, the polarization of the electrolyte can be significant and could dramatically influence the battery performance.^2^ In the present work, we focus on such contributions that enable a more accurate and physical model of lithium-ion batteries.

As an important case of analysis, we have taken the well-studied lithium-ion battery with its electrolyte composed of a lithium salt (LiPF_6_) and two organic carbonates as cosolvents, ethylene carbonate (EC) with either diethyl carbonate (DEC) or dimethyl carbonate (DMC). These components are typical in lithium battery research and in commercial batteries^3^ and have not earlier been rigorously examined as an electrolyte mixture of independent components.

The polarization contributions are given by the gradient in the electric potential, ∇φ, between two lithium metal electrodes. Here, we will only consider one-directional transport, i.e., *d*φ/*dx*, but this can be extended to two- or three-dimensional systems. We express the gradient in electric potential, ∇φ, using nonequilibrium thermodynamics.^4^ By choosing the cosolvent EC as frame of reference, we obtain
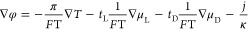
1where the first term on the right-hand side is proportional to the temperature gradient, ∇*T*, via the Peltier coefficient π over the temperature *T* and Faraday’s constant *F*. The second and third contributions contain the gradient in chemical potential of lithium salt, ∇μ_L,*T*_, (L is used as short-hand notation for the lithium salt here), and the gradient in chemical potential of the cosolvent, ∇μ_D,*T*_ (where D in this work can be DEC or DMC). Both gradients are evaluated at constant temperature, as indicated by subscript *T*. The transference coefficient of component *i*, *t*_*i*_, is defined as the mass flux of *i* at a constant composition and temperature over the electric current density. It can be determined from, e.g., Hittorf experiments.^5^ The ohmic potential drop is the fourth term, where the electric current density *j* is multiplied with the inverse electrolyte conductivity, 1/κ.

The expression 1 originates in the entropy production of the cell, when neutral components are used to describe the entropy production. We describe transport in the bulk electrolyte under polarization conditions^6^ by
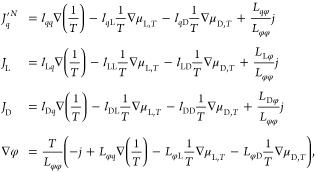
2where  and *L*_*ij*_ are Onsager coefficients for the electrolyte mixture under different conditions. The large and small coefficient symbols are related by
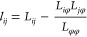
3where the coefficient *L*_φφ_ = *κT*. *J*_*q*_^′*N*^, *J*_L_, *J*_D_, and *j* are the measurable heat flux, mass fluxes of salt and cosolvent, and electric current density, respectively. The measurable heat flux and electric current density do not depend on the frame of reference. The mass fluxes do. They are here measured relative to EC. *L_ij_* are coefficients for transport of heat, mass, and charge. The coefficients  refer to diffusion in the absence of an electric current.^5^ We observe that the value of ∇φ is equal to the ohmic potential drop in the absence of gradients in the composition and temperature. Transport in the electrolyte can be described in two ways, by the mixed (ions and solvents) or by the neutral (salt and solvents) component scenario.^6^ The transport coefficients in the mixed component scenario (Λ^*ij*^) are obtained directly from the fluctuation dissipation theorems using molecular dynamics (MD) simulations, see further explanation in the Supporting Information and in ref (6). The coefficients of the neutral component scenario, used in the equations above, can be obtained by converting the set of Λ^*ij*^ using the Rules for Coupling of Fluxes.^6^

The ability to accurately compute the potential profile in eq 1 has so far been much hampered by a lack of data. Properties of binary electrolytes are well studied e.g., by Newman et al.,^1,7^ but most lithium-ion battery electrolytes are ternary or even quaternary mixtures with more than one solvent. The transport properties of such complex mixtures are not fully known, and coupling of transport phenomena is therefore often neglected.^8^ Assumptions have not been controlled, and little distinction has been made between descriptions with one or more solvent components. The mixture of solvents has often been considered as one component.^9−12^ Recent studies indicate that solvent components separate in the cell.^13,14^ The structure of the ternary electrolyte is, therefore, central for the description of components and their transport properties.

The aim of this work is thus to determine a complete set of transport properties obtained from MD simulations that enable us to compute all contributions to the electric potential at the stationary state. The transport coefficients are needed for battery modeling purposes. The frame of reference for *t*_*i*_ and the choice of components *i* will prove essential as the magnitude of the terms vary with the choice of the frame of reference.^15,16^ The value of eq 1 is, however, independent of the frame of reference. We shall apply a method recently described by Kjelstrup et al., providing new relations for coefficient determinations, the so-called Rules for Coupling of Fluxes.^6^ We start by describing the microstructure of the electrolyte at equilibrium. This is next used as a foundation for explaining thermodynamic and transport properties. A convenient choice of frame of reference will be explained based on the electrolyte microstructure and the diffusion coefficients in different frames of reference. We present and discuss first the effect of diffusion coefficients. The Seebeck coefficient and the heats of transfer will be reported, giving the coupling between the temperature gradient and the electric potential gradient. Finally, all determined parameters are used to estimate the impact of the temperature gradient and solve eq 1 in the stationary state. We can then evaluate how much each term in eq 1 contributes to the cell voltage.

## Results and Discussion

### Equilibrium Structure of the Electrolyte

The structure information reported below suggests that the following exchange takes place in the presence of solvents DEC and EC at equilibrium
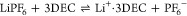


The reaction conveys two ways of viewing the electrolyte: as composed of a mixture of ionic and neutral components (right side) or as a mixture of neutral components only (left-hand side). The transport coefficients of the electrolyte can be formulated using either set of components, and they are connected via the Rules for Coupling of Fluxes, see Kjelstrup et al.^6^ Both sets of components (of mixed and neutral components) were used to find the relevant sets of transport coefficients.

**Figure 1:**
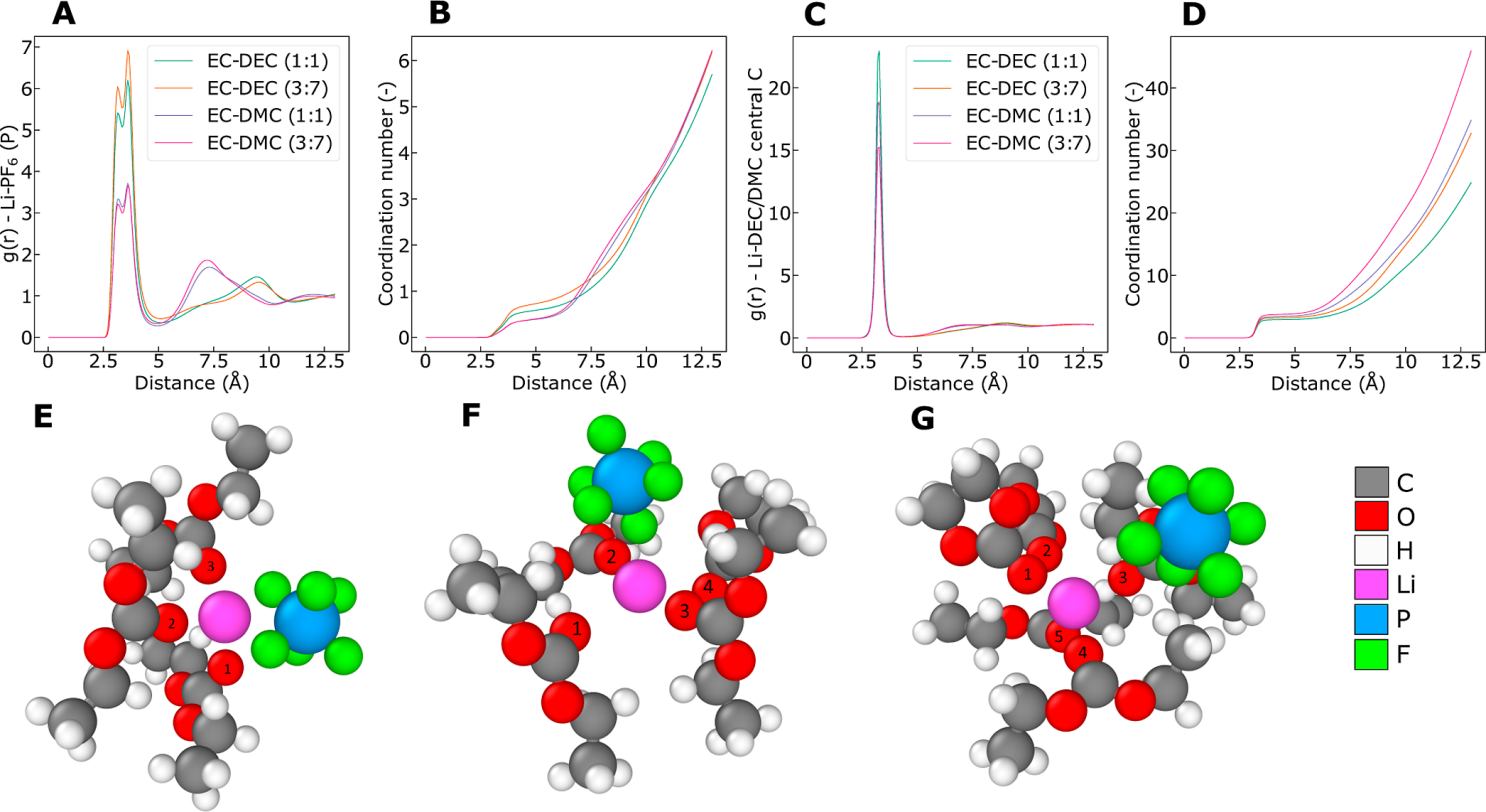
(a) RDFs of Li^+^ and PF_6_^–^ and corresponding (b) coordination numbers as a function of distance. (c) RDFs of Li^+^ and DEC/DMC and corresponding (d) coordination numbers as a function of distance. (e) MD snapshot of Li^+^ coordinated by three DECs and one PF_6_^–^, corresponding to the closest peak of the bimodal RDF of Li^+^ and PF_6_^–^. (f) MD snapshot of Li^+^ coordinated by three DECs, one EC, and one PF_6_^–^, corresponding to the second-closest peak of the bimodal RDF of Li^+^ and PF_6_^–^. (g) MD snapshot of Li^+^ coordinated by four DECs and one EC, the anion is outside the first solvation shell (solvent-separated ion-pair). The coordinating solvent carbonyl oxygen atoms are numbered. Particle colors are shown to the right.

The coefficients of transport for the ternary electrolytes will be understood in terms of the electrolyte structure. We computed the radial distribution function (RDF) for Li^+^ and DEC/DMC and PF_6_^–^ in order to examine the electrolyte structure and the coordination environment of the Li^+^ ions. The results are shown in Figure 1 and the results for Li^+^ and EC are shown in SI, Figure S4. The residence time, i.e., the average time that two species stay together within a specified cutoff distance before parting, provides information about the dynamic properties of the coordination environments.^17−19^ Coordination numbers and residence times are listed in Table 1.

**Table 1:** Coordination in Electrolytes. The Cutoff is Defined as the First Minimum after the First Peak in the RDF, i.e., the First Solvation Shell

electrolyte	pair	coordination number	residence time (ns)	cutoff (Å)
1:1 EC/DEC	Li-EC	1.40	0.42	4.52
1:1 EC/DEC	Li-DEC	2.92 ± 0.01	4.26 ± 0.06	4.45
1:1 EC/DEC	Li-PF_6_	0.59	1.34 ± 0.12	5.08 ± 0.03
3:7 EC/DEC	Li-EC	0.81	0.54	4.52
3:7 EC/DEC	Li-DEC	3.23	3.82	4.45
3:7 EC/DEC	Li-PF_6_	0.74	2.19	5.10
1:1 EC/DMC	Li-EC	1.01	0.18	4.45
1:1 EC/DMC	Li-DMC	3.42	1.87	4.39
1:1 EC/DMC	Li-PF_6_	0.39	0.48	4.97
3:7 EC/DMC	Li-EC	0.59	0.17	4.52
3:7 EC/DMC	Li-DMC	3.74	1.35	4.39
3:7 EC/DMC	Li-PF_6_	0.40	0.46	4.97

Figure 1 shows that each Li^+^ on average is coordinated primarily by the linear carbonates in all electrolytes investigated, i.e., by DEC or DMC. The coordination numbers of DEC and of DMC in the first solvation shell range from 2.9 to 3.2 and 3.4 to 3.7, respectively. The corresponding numbers for EC range from 0.8 to 1.4 and 0.6 to 1.0 in the DEC- and DMC-containing electrolytes, respectively; see Figure S4. Additionally, from Table 1 we see that on average, the Li^+^ spends about an order of magnitude longer time coordinated to DEC/DMC molecules than to EC molecules before changing coordination. All of the solvent molecules are facing Li^+^ by the central carbonyl oxygen. The coordination of Li^+^ in mixed carbonate electrolytes has been a point of discussion in the literature, but no consensus has been reached. Several studies indicate that Li^+^ is preferentially coordinated by EC in electrolyte mixtures of EC and DEC/DMC^20−25^ or that Li^+^ is coordinated equally by EC and DEC/DMC,^26^ but other studies indicate favored coordination by the linear carbonates.^27−29^ There is less than one PF_6_^–^ coordinating Li^+^ on average in all electrolytes. The salt dissociation in the electrolytes containing DMC^30^ is seemingly larger than in DEC. The RDFs of Li^+^ and PF_6_^–^ hint at the presence of ion clusters in the electrolyte. The fractions of ions in the ionic clusters are shown in Table 2. Ions are assumed to be part of a cluster if the interionic distance is less than 5 Å, the distance of the first minimum after the first peak of the Li^+^-PF_6_^–^ (P) RDF. More than 15% of the ions in the 1:1 wt % EC/DEC electrolyte are part of clusters with three or more ions. These clusters are dynamic and relatively short-lived, as indicated by the residence times in Table 1.

**Table 2:** Ionic Clusters in the Electrolytes. Fraction of Free Ions and Fraction of Ions in Different Sized Clusters

electrolyte	free ions	2 ions	3 ions	4 ions	≥5 ions
1:1 EC/DEC (1)	0.49	0.34	0.11	0.04	0.02
1:1 EC/DEC (2)	0.48	0.34	0.11	0.04	0.02
1:1 EC/DEC (3)	0.49	0.34	0.11	0.04	0.02
3:7 EC/DEC	0.37	0.40	0.13	0.06	0.03
1:1 EC/DMC	0.64	0.28	0.06	0.02	0.00
3:7 EC/DMC	0.63	0.30	0.05	0.01	0.00

The equilibrium exchange reaction presented in the start of this section captures these findings. The reaction expresses how DEC or DMC can shield the ions from each other. DMC does this more readily than DEC. The reaction is slightly shifted to the right in the presence of DMC. It is therefore likely that the charge transport involves solvent transport. We will see later that this can be confirmed.

The thermodynamic factors, which describe deviations from ideal mixture theory, were calculated from Kirkwood-Buff integrals (Supporting Information) and are presented in Table 3. A main factor Γ_*ii*_ equal to one and a cross factor Γ_*ij*_ equal to zero means that the mixture is ideal, cf. Simon et al.^31^ The values of the main factors Γ_LL_ and Γ_DD_ are clearly above one in all electrolytes, indicating the presence of repulsive forces. The cross-terms Γ_LD_ and Γ_DL_ are smaller than the corresponding main factors, indicating more attractive forces between polar DEC/DMC molecules and the ions. The thermodynamic factors involving the solvent are sensitive to the solvent composition of the electrolytes.

**Table 3:** Thermodynamic Factors (Γ_*ij*_, L = LiPF_6_, D = DEC or DMC) Calculated Using Concentrations^6^

system	Γ_LL_	Γ_LD_	Γ_DL_	Γ_DD_
1:1 EC/DEC +1 M LiPF_6_	1.68	1.30	5.80	47.0
3:7 EC/DEC +1 M LiPF_6_	1.65	1.42	7.72	58.0
1:1 EC/DMC +1 M LiPF_6_	1.66	0.84	4.89	28.6
3:7 EC/DMC +1 M LiPF_6_	1.63	0.94	7.21	36.8

### From the Barycentric to the Cosolvent Frame of Reference

The frame of reference is central when transport of components in multicomponent mixtures is measured. The transport coefficients depend on the frame of reference. The Onsager coefficients from the simulations were obtained in the barycentric (or wall) frame of reference. The flux–force matrix of the isothermal system in this frame of reference has 10 coefficients, but we can reduce this number, using the fact that the driving forces are dependent through Gibbs–Duhem’s equation, cf. Ref (6). Two possibilities for elimination of driving forces are then possible: EC or DEC. To help in that decision, we provide Onsager coefficients in Supporting Information Table S1 for the barycentric, EC-, and DEC frames of reference.

Consider first *L*^++^ = (0.35 ± 0.04) × 10^–11^ m^2^ s^–1^ in the barycentric frame of reference. Upon transformation to the EC frame of reference, *L*^++^ becomes (0.8 ± 0.1) × 10^–11^ m^2^ s^–1^ and in the DEC frame of reference, *L*^++^ is (0.3 ± 0.1) × 10^–11^ m^2^ s^–1^. The coefficient *L*^++^ is larger when measured relative to EC than to DEC because Li^+^ is less strongly coordinated to EC than to DEC. Both EC and DEC move with respect to the center of mass frame of reference, and they also move relative to one another. To treat the solvent as one component only, as is done in the literature,^32^ means to neglect these relative movements. Furthermore, *L*^––^ is (0.7 ± 0.2) × 10^–11^ m^2^ s^–1^ in the barycentric frame of reference, (1.3 ± 0.2) × 10^–11^ m^2^ s^–1^ in the EC frame of reference, and (1.0 ± 2) × 10^–11^ m^2^ s^–1^ in the DEC reference frame. The PF_6_^–^ anion is weakly coordinated by solvent molecules and moves more independently of the solvent, resulting in a smaller difference between the EC- and DEC frames of reference. The coefficient *L*^ED^, which is present only in the barycentric frame of reference, is negative. This suggests that EC and DEC tend to move away from one another. In fact, EC moves away from all the other components in the barycentric reference frame and apparently is not much directly involved in charge transport. This gives arguments in favor of choosing the cosolvent EC as frame of reference for a reduced set of coefficients.^7,9,10,33,34^ To use a mixture of solvents as the frame of reference gives fewer components transported and less variables. We have chosen to use EC alone as a frame of reference for the mass fluxes. The number of unknown coefficients is reduced from ten to six with this choice. We will have the possibility to study solvent segregation, which has recently been observed experimentally.^13^ The choice for component EC is thus motivated by EC being less involved in structure-making than DEC as well as in the transport of Li^+^ and charge. Note, however, that upon going from the barycentric to a cosolvent as frame of reference, some information about the system is lost, e.g., the correlation of the solvent components EC and DEC, *L*^ED^. Moreover, if the motion of the cosolvent chosen as frame of reference is unknown, the interpretation of the transport coefficients becomes less transparent.^35,36^

### Coefficients for Isothermal Diffusion

As mentioned, the electrolyte can be equivalently described by the mixed component or the neutral-only component scenario. At isothermal conditions, molecular simulations naturally produce transport coefficients in the mixed component scenario. But operationally defined, experimentally obtained properties are usually related to neutral components.^5,37^ The Rules for Coupling of Fluxes provide links between the two scenarios and thus between simulations and experiments. The set of transport coefficients of the neutral component scenario is our target, to be used for thermodynamic modeling of the battery electrolyte.

#### Onsager Coefficients

The Onsager coefficients for the mixed component scenario obtained from the fluctuation–dissipation theorems, as shown in the Supporting Information, are presented in Table 4. The Onsager coefficients for the neutral components scenario were computed from these to finally give the electrolyte conductivity plus the transference coefficients for the salt and the cosolvent in the 1:1 EC/DEC with 1 M LiPF_6_ electrolyte. The last properties were obtained using the Rules for Coupling of Fluxes^6^ and are presented in the lower part of Table 4.

**Table 4:** Diffusion Coefficients of the 1:1 wt.% EC/DEC + 1 M LiPF_6_ Electrolyte in the Mixed Component Scenario, Derived from Equations in the Supporting Information and ref (6)., and Converted to the EC and DEC Frames of Reference. Transference Coefficients, *t*_L_, *t*_D_, and *t*_E_, and Transport Numbers, τ_+_ and τ_–_, are Dimensionless. The Coefficients in the Mixed Scenario *L*^*ij*^ Have Dimension m^2^ s^–1^. The Dimension Needed for eq 2 Is Obtained by Multiplication with *c*/*R* and These Coefficients Are Shown in the Rightmost Column

frame of reference	EC	DEC	EC
coefficient	value ×10^–11^ m^2^ s^–1^	value ×10^–11^ m^2^ s^–1^	value ×10^–9^ K mol^2^ J^–1^ m^–1^ s^–1^
*L*^++^	0.8 ± 0.1	0.3 ± 0.1	12.1 ± 0.9
*L*^––^	1.3 ± 0.2	1.0 ± 0.2	18.7 ± 3.0
*L*^+–^	0.56 ± 0.05	0.2 ± 0.1	8.4 ± 0.7
*L*^D+^	2.4 ± 0.2		36.4 ± 3.2
*L*^D–^	1.8 ± 0.2		27.5 ± 2.7
*L*^DD^	11.3 ± 1.3		167.8 ± 18.7
*L*^E+^		0.1 ± 0.3	
*L*^E–^		0.9 ± 0.1	
*L*^EE^		20.3 ± 2.3	
*L*_φφ_ = *κT*	(69.3 ± 10.4) K S m^–1^		
*L*_φL_	(−7.5 ± 2.3)×10^–4^ K mol C J^–1^ m^–1^ s^–1^		
*L*_φD_	(6.4 ± 2.5)×10^–4^ K mol C J^–1^ m^–1^ s^–1^		
κ = *L*_φφ_/*T*	(0.23 ± 0.03) S m^–1^		
*t*_L_	–0.97 ± 0.12	–1.17 ± 0.06	
*t*_D_	0.90 ± 0.46		
*t*_E_		–1.21 ± 0.62	
τ_+_	0.28 ± 0.09	0.12 ± 0.04	
τ_–_	0.72 ± 0.09	0.88 ± 0.04	

We see from the table that *L*^––^ is larger than *L*^++^, which means that PF_6_^–^ will move faster than Li^+^. This is also reflected in the low Li^+^ transport number (τ_+_) of 0.28. This value is comparable to experimental values for the Li^+^ transport number in EC/DEC + LiPF_6_ electrolytes reported by, e.g., Lundgren et al.^11^ and Landesfeind and Gasteiger.^10^ These studies report transport numbers relative to the solvent mixture (as most experimenters do), while our results are relative to the EC. In other words, we assume that *J*_EC_ = 0. Notably, τ_+_ in the DEC frame of reference is only 0.12 as Li^+^ and DEC move together. A positive *L*^+–^ means that the cation and anion movements are positively correlated; i.e., they tend to move together and reduce ionic conductivity. This is reflected in the RDF values of Li^+^ and PF_6_^–^ in Figure 1a and in Tables 1 and 2. The *L*^D+^ is quite large and positive, which means that there is a strong tendency for correlated motion of Li^+^ and DEC, as reflected in the corresponding RDF in Figure 1c and the residence times. Interestingly, *L*^D–^ is positive and significant but smaller than *L*^D+^, so DEC will mostly follow Li^+^. Generally, the coupling coefficients, *L*^*ij*^, are of the same order of magnitude as the main coefficients, *L*^*ii*^. They should hence not be neglected, as is now common. A large *L*^DD^ indicates that DEC is moving quickly relative to EC, indicating again that the assumption of the solvent mixture moving as one component is not true. Already from the results under isothermal conditions, we see that gradients in salt concentration and solvent composition will evolve in the electrolyte during charge or discharge of the battery. This will affect the battery voltage and will be demonstrated later. The transference coefficients *t*_L_ and *t*_D_ define the amount of salt and DEC transferred when 1 F of positive charges is passing the electrolyte from left to right.

In particular, 0.90 mol of DEC is transferred with the passage of 1 F of electric charge through the electrolyte. Consequently, DEC will move toward the cathode side, when measured relative to EC. This finding is not in agreement with a recent experimental study by Wang et al.^13^ They showed that the linear carbonate cosolvent, ethyl methyl carbonate (EMC), in an electrolyte mixture with EC and LiPF_6_ accumulated on the anode side upon passage of current. If the Li^+^ were primarily coordinated by EC molecules in the simulations, we expect that EC would follow Li^+^ and accumulate on the cathode side, and the linear carbonate DEC would move toward the anode side to fill the remaining void, as in the experiment. This deviation between our simulations and experimental results points to a potential inaccuracy of the force field that in reality, Li^+^ is primarily coordinated by the cyclic carbonate EC and not by the linear carbonate. Our results for the salt transference coefficient mean that salt accumulates on the anode side. The electrodes are reversible to Li^+^ ions and produce 1 F of lithium ions in the adjacent electrolyte, while only a fraction of 0.28 leaves the electrolyte chamber.

The ionic conductivity of the simulated electrolyte is 0.23 S m^–1^, which is below the measured value of about 0.8 S m^–1^ by Lundgren et al.^11^ However, even though the absolute values of the transport coefficients are lower than the experimentally measured values, the ratios expressed as transference coefficients are seemingly correct. The *L*_φL_ and *L*_φD_ coefficients describe how the components move in the electric field or respond to the net electric current. Their sign gives the direction of transport, positive when the movement follows positive charges and vice versa for the opposite sign. The transference coefficient is given by the ratio of this coefficient and the ionic conductivity multiplied by Faraday’s constant.^6^

#### Fick’s Diffusion Coefficients

Fick’s diffusion coefficients are more frequently measured than the Onsager coefficients since there is easier access to gradients in concentration than to the gradients in chemical potential. The set of Fick’s coefficients describes the same reality as the Onsager coefficients. The two sets are therefore related by entropy production invariance. The Fick’s law coefficients were computed using the equations in ref (6), and the results are shown in Table 5. For example, *D*_LL_ is the diffusion of salt due to a concentration gradient of salt, and *D*_LD_ is the diffusion of salt due to a concentration gradient of DEC. The symmetry of the Onsager coefficient matrix is no longer present in Fick’s diffusion coefficients, meaning that four rather than three coefficients are needed. There is also no requirement that Fick’s main diffusion coefficients must be positive like for the Onsager main coefficients. The thermodynamic factor relates the chemical potential gradient and concentration gradient and is used to convert Onsager coefficients to Fick’s diffusion coefficients. The values of the thermodynamic factors depend on the ensemble conditions and concentration units used. Performing the conversion increases the potential error and the method for computing thermodynamic factors could be a source of ambiguity.^6^

**Table 5:** Diffusion Coefficients of the Isothermal 1:1 wt.% EC/DEC + 1 M LiPF_6_ Electrolyte in the EC Reference Frame. The Six Top Values for the Neutral Component Scenario Are Computed from the Coefficients in Table 4 Using the Generalized Transport Model and Equations in ref (6). The Four next Values Are Diffusion Coefficients from Fick’s Extended Law, Equations in ref (6). The Four Bottom Values Are the Self-Diffusion Coefficients. Conditions Are the Same as for Table 4

coefficient	value ×10^–11^ m^2^ s^–1^	value ×10^–9^ K mol^2^ J^–1^ m^–1^ s^–1^
*L*_LL_	1.3 ± 0.2	18.7 ± 3.0
*L*_DL_	1.8 ± 0.2	27.5 ± 2.7
*L*_DD_	11.2 ± 1.3	167.8 ± 18.7
l_LL_	0.74 ± 0.04	11.0 ± 0.6
l_DL_	2.3 ± 0.1	33.8 ± 1.9
l_DD_	10.8 ± 1.3	161.0 ± 19.2
*D*_LL_	52.3 ± 2.6	
*D*_LD_	309.4 ± 16.9	
*D*_DL_	222.8 ± 22.4	
*D*_DD_	1453.5 ± 170.2	
*D*_Li^+^_	7.2 ± 0.2	
*D*_PF_6_^–^_	12.4 ± 0.2	
*D*_D_	11.6 ± 0.1	
*D*_E_	22.1 ± 0.1	

The advantage of Fick’s diffusion coefficients is that they can be compared to experimental results. Lundgren et al.^11^ obtained Fick’s diffusion coefficients in electrolytes containing LiPF_6_ in EC/DEC by measuring the relaxation of the open circuit potential after applying a small current for a certain time through the electrolyte sandwiched by Li electrodes. They calculated an effective diffusion coefficient of the salt in the mixed solvent frame of reference for the mixture of 1.5 × 10^–10^ m^2^ s^–1^ for a salt concentration of 1 M. This value is of the same order of magnitude as the calculated Fick’s diffusion coefficients in Table 5. Unlike in the above-mentioned experiment, simulations give four Fick’s diffusion coefficients in a ternary mixture. The four coefficients are not separable in the experiments, so the experimental result can be viewed as an effective diffusion coefficient composed of four contributions. The disadvantage of a description using Fick’s coefficients is that the driving forces are not fully captured by the concentration gradients.

#### Self-Diffusion Coefficients

Self-diffusion coefficients of all components are also provided in Table 5. These values can be compared to measurements, e.g., to nuclear magnetic resonance (NMR) spectroscopy. Hayamizu^38^ measured the self-diffusion coefficients of all components of a 1 M LiPF_6_ in 4:6 EC/DEC electrolyte at 303 K. The self-diffusion coefficients of EC, DEC, Li^+^, and PF_6_^–^ were (3.5, 3.60, 1.70 and 2.61) × 10^–10^ m^2^ s^–1^. Notably, EC and DEC move almost equally fast in the experimental setup while in our simulations, EC moves faster than DEC. The experimental and simulated values can only be expected to be of the same order of magnitude.

#### Composition Dependence

The transport coefficients under isothermal conditions for the 1:1 EC/DMC with the 1 M LiPF_6_ electrolyte are presented in Table 6. All transport coefficients in the 1:1 EC/DMC system are larger than the corresponding coefficients in the 1:1 EC/DEC electrolyte (Table 4). The resulting ionic conductivity of 1:1 EC/DMC is about twice as high as in the 1:1 EC/DEC electrolyte due to faster dynamics and improved salt dissociation with the shorter DMC molecule.

**Table 6:** Transport Coefficients of the 1:1 wt.% EC/DMC + 1 M LiPF_6_ Electrolyte in the Mixed Component Scenario Using the EC Frame of Reference. Transference Coefficients, *t*, and Transport Numbers, τ, Are Dimensionless

coefficient	value ×10^–11^ m^2^ s^–1^	value ×10^–9^ K mol^2^ J^–1^ m^–1^ s^–1^
*L*^++^	1.1	18.8
*L*^––^	1.9	33.4
*L*^+–^	0.6	10.9
*L*^D+^	4.2	72.6
*L*^D–^	3.2	54.8
*L*^DD^	26.9	464.5
κ	0.48 S m^–1^	
*t*_L_	–0.99	
*t*_D_	0.78	
τ_+_	0.26	

Transport coefficients for the 3:7 EC/DEC and EC/DMC systems are provided in Tables S2 and S3. By increasing the concentration of the linear carbonate, we generally obtain larger transport coefficients and increased electric conductivity. There are no dramatic changes in the transference coefficients, however.

### Thermal Polarization of the Electrolyte

We have discussed above that concentration polarization takes place in the isothermal electrolyte. In the presence of a temperature gradient, we need to include thermal polarization. When a temperature difference is applied or arises between the electrodes of an electrochemical cell, we can observe a distribution of components in the thermal field (a Soret effect) as well as migration of charges to produce a cell voltage (a Seebeck effect). The two effects are superimposed. Both effects affect the cell voltage.

#### Seebeck Coefficients

The Seebeck coefficient is defined as the cell potential difference measured by two identical electrodes caused by an applied temperature difference under reversible conditions with a uniform electrolyte composition. The cell potential, obtained by integrating eq 1, includes also a Soret effect via the change in the chemical potential gradients in this equation. Through the Onsager reciprocal relations, the Peltier heat of the electrode surface and from it the Peltier coefficient of the electrolyte can be computed from the Seebeck coefficient, cf. eq 11 in the Supporting Information.

**Figure 2:**
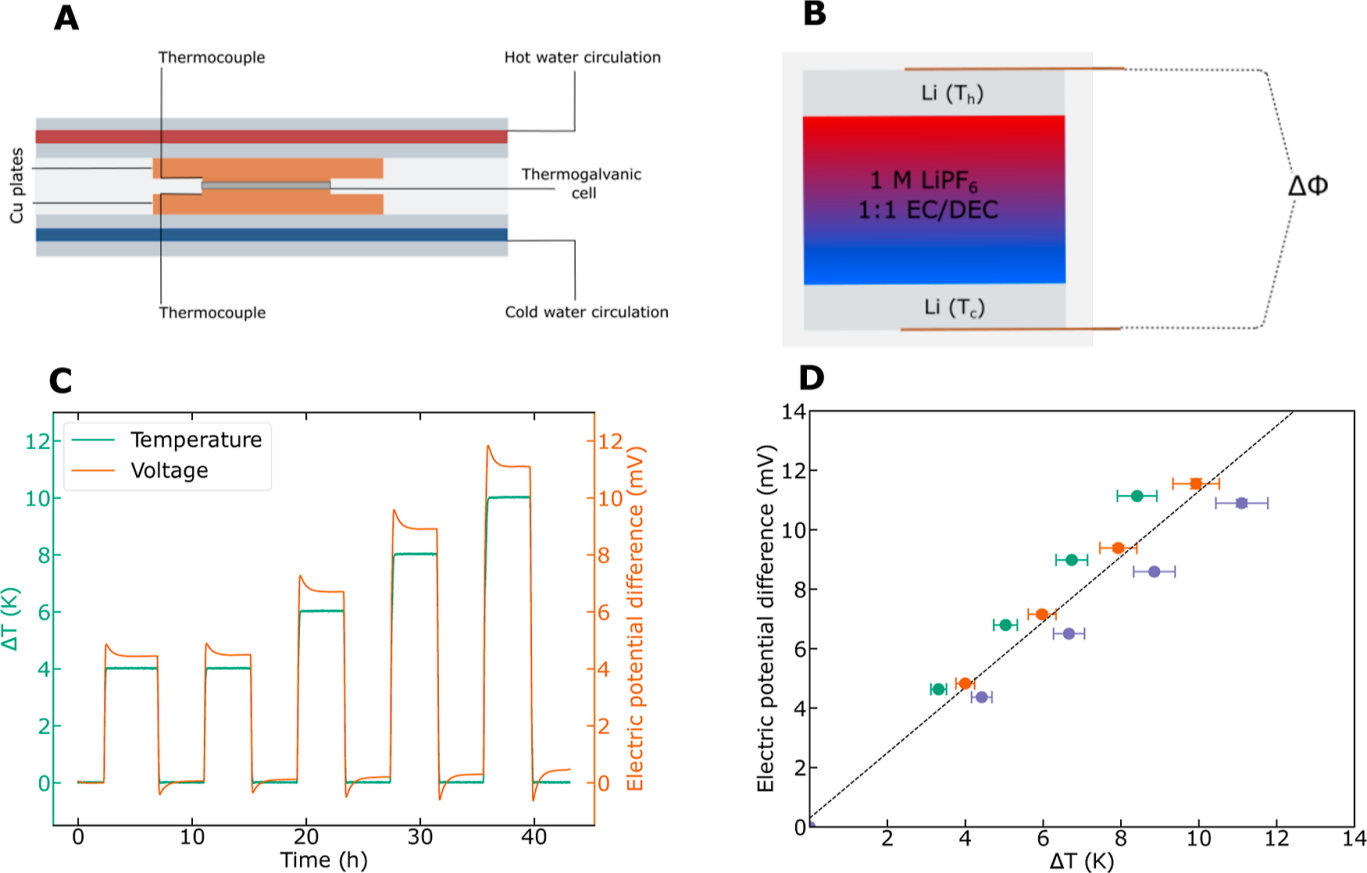
(a) Schematic diagram of the experimental setup for measuring Seebeck coefficients in electrolytes. (b) Close-up view of the electrolyte between hot and cold Li electrodes. (c) Temperature difference between Li electrodes and electric potential difference as a function of time in the 1 M LiPF_6_ in 1:1 EC/DEC electrolyte. (d) Electric potential difference as a function of temperature difference in three parallel experiments on the 1 M LiPF_6_ in 1:1 EC/DEC electrolyte. The slope of the linear line is the Seebeck coefficient.

The Seebeck coefficient was measured in a symmetric Li–Li cell. In these experiments, the cell was sandwiched between two copper plates; see Figure 2a for a sketch. The high temperature at the top copper plate and the low temperature at the bottom copper plate were controlled by thermostated water flowing from a water reservoir to the copper plate in question. Each copper plate with its electrode was insulated from the surroundings. The temperature difference between the electrodes was measured or computed from a calibration experiment and the temperature difference of the copper plates, cf. Figure 2b. See ref^39^ for more details. The electric potential difference was measured as a function of the temperature difference between the copper plates,^4,40^ see Figure 2c,d.

Figure 2d shows a typical plot of Δφ as a function of Δ*T* for the present choice of electrolyte. From the slope of the curve in Figure 2d, we computed the Seebeck coefficient first due to thermal polarization at the start of the experiment (*t* = 0), when the electrolyte is still homogeneous. The slope that we derived from three measurements gave the value 1.15(20) mV K^–1^ at 300 K for the 1 M LiPF_6_ in 1:1 (vol) EC/DEC electrolyte. The Seebeck coefficient with the 1:1 wt % EC/DMC electrolyte was determined to 1.1(1) mV K^–1^ at 300 K based on 14 measurements,^39^ equal to the DEC-containing electrolyte within experimental uncertainty. This value translates into a Peltier heat of 300 K × 1.15 × 10^–3^ × 1 × 10^5^ V C K mol = −34.5 kJ mol^–1^. This is the reversible heat that is generated or absorbed at the electrode surface, here being a source at the anode boundary and a sink at the cathode boundary during operation of the battery. By subtracting the electrode contribution to the Peltier heat (*S*_Li_^0^ = 29 J K^–1^ mol^–1^), we computed the Peltier coefficient of the electrolyte to be 300 K × 0.86 × 10^–3^ × 1 × 10^5^ V C K^–1^ mol^–1^ = −24.7 kJ mol^–1^ using eq 12 in the Supporting Information, employing a Seebeck coefficient for the bulk electrolyte of 0.86 mV K^–1^.

We shall see below that this Seebeck coefficient has a small contribution from the Soret effect (the heat of transfer of the salt and of DEC is small). We see from Figure 2c that the temperature difference establishes itself within minutes and that the potential difference responds uniquely to the applied temperature difference. In the present case, the initial time value did not change significantly over time, giving a first indication that the Soret effect was indeed small. The prediction was verified below.

#### Soret Effect

Nonequilibrium molecular dynamics (NEMD) simulations gave results for Soret equilibrium, when the thermal driving force balances the chemical driving forces or the gradients in mole fraction of the different components. The balance of forces occurs at the stationary condition (*t* = *∞*) and provides the impact of thermal polarization via gradients in chemical potential, which further adds to eq 1.

**Figure 3:**
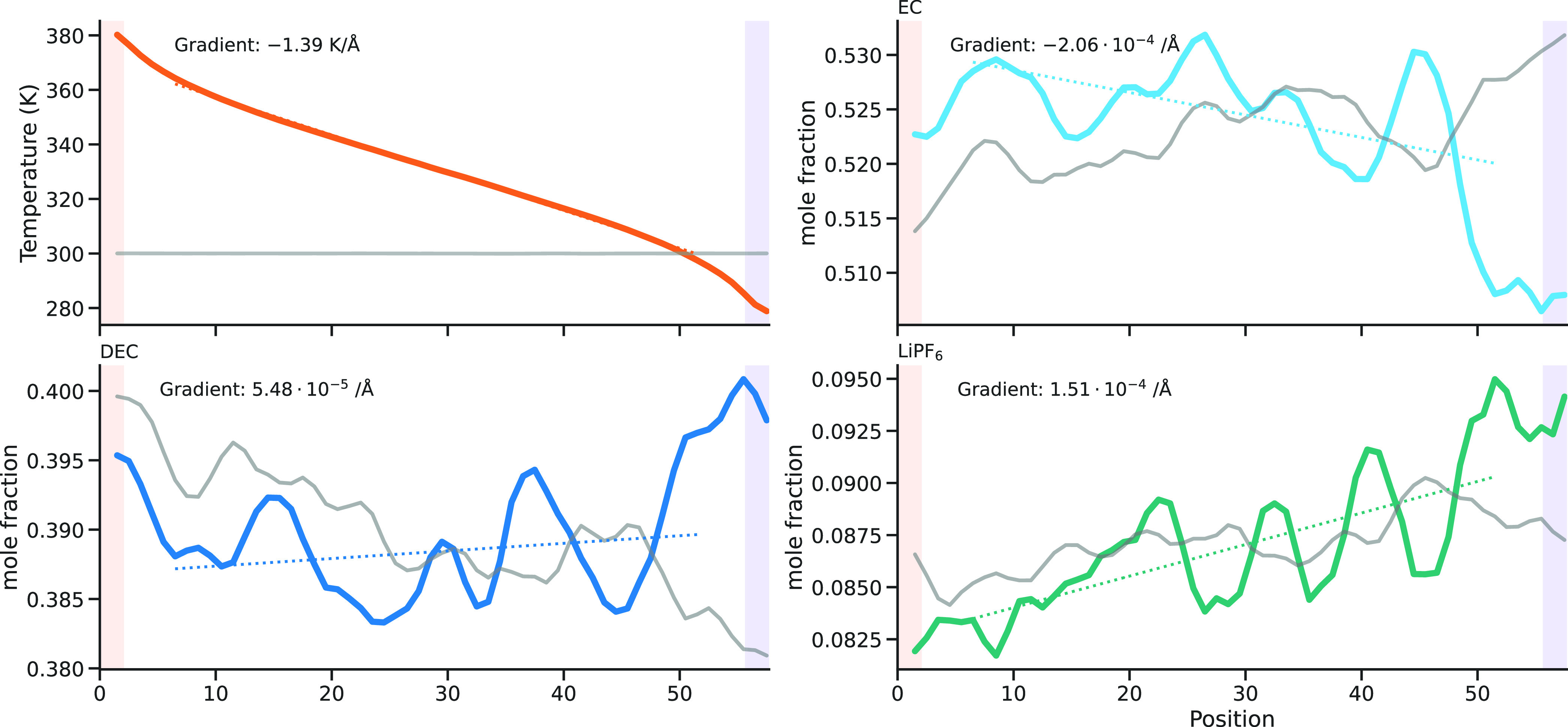
Profiles from the NEMD simulations of the 1 M LiPF_6_ in the 1:1 EC/DEC system for the temperature and the mole fractions of EC, DEC, and LiPF_6_. The gray lines show the profiles from corresponding equilibrium simulations, and the dotted lines show linear fits to the profiles used to determine the gradients. The thermostated regions are highlighted as blue (cold) and red (hot).

We calculated the chemical potential gradients and the heats of transfer by eqs 10 and 9 in the Supporting Informtion. The gradients (and the accompanying heat flux) are given in Table 7 and example profiles are shown in Figure 3. The thermal conductivity (calculated from the heat flux and temperature gradient for the sake of completeness) is approximately 0.2 W K^–1^ m^–1^ in all cases. We find (Table 7) that the heat of transfer of the salt, *q*_L_^*^, is small (about 1 to 2 kJ mol^–1^), which supports the fact that the first terms of π in eq 12 in the Supporting Information dominate (Δφ/Δ*T*)_*j*=0_. The heat of transfer of component DEC, *q*_D_^*^, is even smaller than *q*_L_^*^ (≤0.3 kJ mol^–1^) and is not shown in Table 7. The corresponding composition profiles in Figure 3 do not deviate significantly from the equilibrium profiles obtained without a temperature gradient.

**Table 7:** Heat Flux and Gradients in Mole Fraction (*x_i_*) from the NEMD Simulations. The Value of *q*_L_^*^ Is Evaluated at the Mean Temperature in the NEMD Simulations (330 K). The Thermodynamic Factors Γ_*Ij*_^*x*^ Used to Calculate *q*_i_^*^ Are Provided in Table S1

	*J*_*q*_^′^×10^–9^	∂*T*/∂*z*	∂*x*_*i*_/∂*z* × 10^4^ (Å^–1^)				*q*_L_^*^
system	(W m^–2^)	(K Å^–1^)	LiPF_6_	DEC	EC	DMC	(kJ mol^–1^)
1:1 EC/DEC	2.8	–1.39	1.5	0.55	–2.1		1.6
3:7 EC/DEC	3.0	–1.45	1.3	–0.66	–0.69		1.1
1:1 EC/DMC	2.8	–1.36	1.01		0.42	–1.4	1.2
3:7 EC/DMC	3.0	–1.43	2.1		0.50	–2.6	2.2

The Soret effect is reflected in the time-dependence of the electric potential difference, when the force balance of thermal and chemical forces establishes itself; see Figure 2c. We see that the Soret effect seems to give a negative contribution to the electric potential difference. The observed effect is the sum of products of heat of transfer and transference coefficient of the independent electrolyte components times the inverse temperature.^40^ The difference in the initial and stationary state values of the Seebeck coefficient was here smaller than the experimental uncertainty; therefore, no value could be extracted from the experimental data. A contribution to the relaxation from phase-change phenomena in the electrode has also been suggested.^39^ For our purpose, to compute the thermal polarization, we conclude that the Soret coefficient or the heat of transfer in the present case is so small that it has a negligible impact on the gradients in chemical potential and therefore on the thermal polarization. In a good approximation, the thermal polarization is due to the Seebeck coefficient alone. The contribution can simply be added to the concentration polarization of the cell voltage.

### Total Polarization of Lithium-Ion Battery Electrolytes

We can now return to the question raised upfront; how large can we expect the concentration polarization and the thermal polarization to be in a lithium-ion battery, i.e., what are the contributions on the right-hand side of eq 1 to the battery voltage? The coefficients that entered the equation have now been defined and determined.

Consider first the events that take place when an electric current is passing through the isothermal electrolyte. Charge is transported and solvent DEC/DMC (D) is carried along, leading to the buildup of a gradient in chemical potential of both salt along with accumulation of cosolvent D. Mass transfer, or reaction heat sinks and sources, will eventually also lead to a temperature gradient. We are interested in both types of polarization, and can now compute them at the stationary state operation, when *J*_D_ = 0 and *J*_L_ = 0.

#### Concentration Polarization

Consider first isothermal conditions: ∇*T* = 0. We apply eqs 2 and 3 and use one of the conditions to express the other chemical potentials.
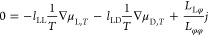
4
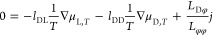
5
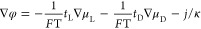
6We introduce the transference coefficients into the flux conditions, and obtain
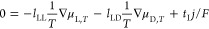
7
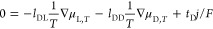
8The last equation is used to express ∇μ_L,*T*_, which we introduce in the equation above to give
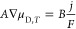
9where
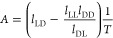
10and
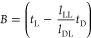
11The solution for the isothermal electric potential difference of the cell with two lithium-reversible electrodes at stationary state becomes
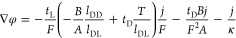
12

The equation describes the three types of losses described above, in electric potential on the right-hand side. All of them are proportional to the electric current density, *j*. The last term on the right side represents the potential ohmic loss; the central term represents loss due to a gradient in D (concentration polarization due to D), and the first term on the right side is due to the accumulation of salt at the anode, producing a peak in the chemical potential gradient of salt at this location. At the stationary state, the isothermal electric potential gradient depends only on the transport properties. This is the solution in the absence of a temperature gradient. In the presence of a temperature gradient, there is one more term, here computable from the Seebeck coefficient; see below.

**Figure 4:**
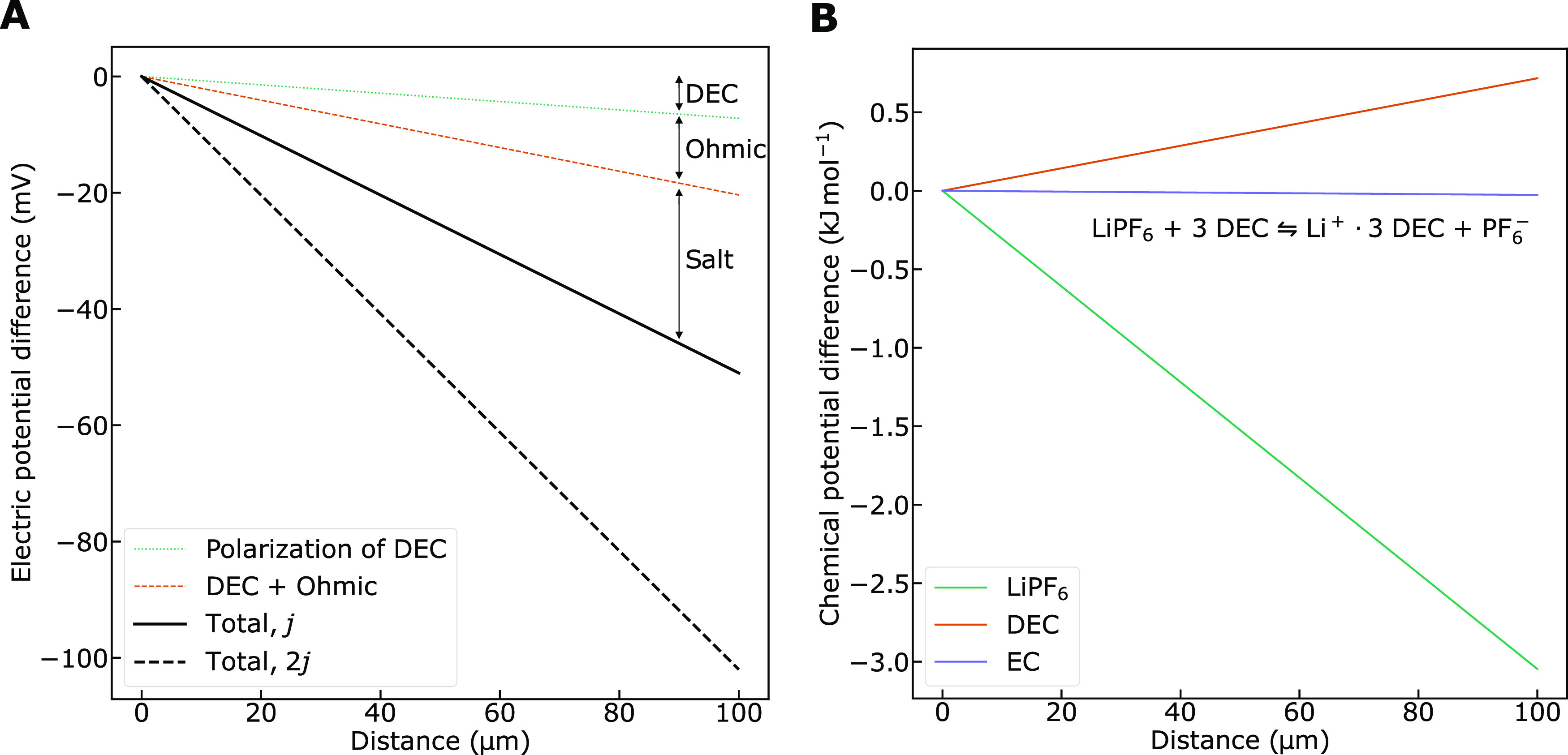
(a) Isothermal electrolyte contributions to electric potential difference in the stationary state as a function of distance from the anode during discharge. Contributions are shown for a current density corresponding to a discharge time of 1 h (1 C). The total polarization for a current density corresponding to a discharge time of 0.5 h (2 C) is also shown. Interface resistances are not taken into account. (b) Chemical potential gradients of the three components in the stationary state as a function of distance from the anode during discharge. The chemical potential gradient of LiPF_6_ is negative and it is slightly positive for DEC. In the EC frame of reference, the chemical potential of EC is constant. Only relative differences matter, i.e., the starting point is arbitrary. Interface resistances are neglected.

For the relevant concentration polarization, we know all coefficients involved and can compute *A* and *B* in the equation above. Their values in the different electrolytes are given in Table S4. The contributions to cell voltage from salt polarization, polarization of solvent D, and ohmic loss in the different electrolytes are presented in Table 8. They are also visualized in Figure 4a.

**Table 8:** Potential Contributions to Cell Voltage in the Isothermal Case

	1/κ	tDF2BA	tLF2(−BAlDDlDL + tDTlDL)
electrolyte	(Ω m)	(Ω m)	(Ω m)
1:1 EC/DEC	4.4 ± 0.7	2.4 ± 1.7	10.2 ± 4.6
3:7 EC/DEC	4.0	0.8	9.3
1:1 EC/DMC	2.2	0.7	5.3
3:7 EC/DMC	1.9	0.7	3.3

The equations and data presented enable us to evaluate the effect of concentration gradients on battery performance under operation. In order to obtain numerical insights, the typical current density *j* = 30 A m^–2^ used by Spitthoff et al. is considered.^41^ This is a current density that can be expected when a fully charged cell is discharged within an hour (1 C rate). The current density gives *j*/*F* = 3 × 10^–4^ mol m^–2^ s^–1^. We are now ready to calculate the various contributions to the cell voltage under isothermal conditions.

The conductivity of the 1:1 EC/DEC electrolyte is 0.23 S m^–1^ (from Table 4), resulting in an ohmic voltage drop of 132 V m^–1^. The distance between the electrode interfaces is given by the separator thickness, which is about 20 μm.^42^ In addition, the electrodes in typical commercial batteries are typically 50 to 60 μm thick.^43^ For the present calculations, we assume a total distance between the electrodes of 100 μm, which is relevant for research cells. Electrodes and the separator are soaked in the electrolyte.^43^ We did not evaluate the effect of porous electrodes and separator in this work but note that they will increase the concentration polarization.^2,44^ This analysis assumes flat and thin Li electrodes. A gradient of 132 V m^–1^ gives a difference of 13.2 mV over 100 μm. Other contributions are of the same order of magnitude. Tables 4 and 5 give for the 1:1 EC/DEC electrolyte, *t*_L_ = −0.97, *t*_D_ = 0.90 and the diffusion coefficient ratio , giving *B* = −1.3. The term *t*_D_ adds to the potential drop. The coefficients of Table 4 have the common factor ×10^–9^ K mol^2^ J^–1^ m^–1^ s^–1^. With this factor, we obtain *A* = −6.3 × 10^–11^ mol^2^ J^–1^ m^–1^ s^–1^ for the 1:1 EC/DEC electrolyte. The gradient in chemical potential of D becomes *Bj*/*AF* = 7.17 × 10^6^ J mol^–1^ m^–1^, which means that there is a 717 J mol^–1^ difference in chemical potential of DEC over 100 μm. The difference amounts to 7.2 mV over this distance. The chemical potential gradients of the three components are displayed in Figure 4b. Polarization of the salt contributes between two and three times more to the voltage than the ohmic resistance. Polarization of component DEC/DMC contributes more than 10% of the total potential contributions, which is in accordance with a recent experimental study on the overpotential due to solvent polarization.^45^

We have thus computed the first, second, and third terms on the right-hand side of eq 12 to combine to (−132–72–306) V m^–1^ = −510 V m^–1^. This gives an electric potential difference at the stationary state of 51.0 mV over the distance between the electrodes (100 μm) in the 1:1 EC/DEC electrolyte for a current density of 30 A m^–2^, disregarding all effects due to electrodes and the separator, see Figure 4.

The potential ohmic loss is smaller in DMC-containing electrolytes due to their higher ionic conductivity. The potential contribution due to polarization of the salt in the DMC containing electrolytes is about half of the corresponding values in the DEC containing electrolytes. The relative contribution from component DMC is smaller than the contribution from component DEC.

#### Thermal Polarization

In this investigation, the Seebeck coefficient was 0.86 mV K^–1^ in both 1:1 EC/DEC/DMC electrolytes, while the Soret coefficient gave a negligible contribution to the cell potential at the stationary state. The thermal polarization in volt at stationary state is therefore equal to the Seebeck coefficient times the temperature gradient and the electrolyte thickness. A difference of 40 K is used here, motivated by accelerated aging experiments with externally applied thermal gradients,^46^ where a severe increase in lithium plating was observed for a battery cycled under a thermal gradient. Knowledge of the temperature difference across the electrolyte is necessary to determine thermal polarization. This is difficult to measure directly due to the very short distance (∼20 μm) between the anode and cathode interfaces in a battery cell. Moreover, the temperature measurement itself could potentially influence the result. Our selected temperature difference of 40 K is likely too large for normal battery operation but enables a calculation of the thermal polarization in the special case of an applied interelectrode thermal force. The contribution due to a temperature difference between the electrode surfaces to the electric potential is important but is not taken into account here.^41^ The results are compared to the other contributions in Table 9. With DEC as the cosolvent, the total polarization amounts to 85.4 mV under these operating conditions. The corresponding value for DMC is 59.1 mV. This is 30% lower compared to when DEC is used and gives a clear advantage to DMC. This cosolvent leads to smaller polarization of salt and component D and a smaller ohmic loss. A concentration variation of EC/DEC/DMC from 1:1 to 3:7 will reduce the ohmic loss, salt polarization, and polarization of component DEC/DMC.

**Table 9:** Electrolyte Potential Contributions to Cell Voltage Assuming a Current Density of 30 A m^–2^ (1 C Rate), 100 μm Distance between Electrodes and Temperature Difference of 40 K between Flat and Thin Electrodes (Average Temperature 300 K). Conductivity and Transference Coefficients Are Assumed to Be Constant in This Temperature Range. Interface Effects Are not Considered

	ohmic loss	polarization of D	salt polarization	thermal polarization	sum
lectrolyte	(mV)	(mV)	(mV)	(mV)	(mV)
1:1 EC/DEC	13.2	7.2	30.6	34.4	85.4
3:7 EC/DEC	12.0	2.3	27.8		
1:1 EC/DMC	6.7	2.0	16.0	34.4	59.1
3:7 EC/DMC	5.6	2.0	9.8		

The potential contributions to the cell voltage reported in Table 9 likely depend on the temperature. The temperature dependence of the transference coefficients and the conductivity was examined by conducting equilibrium simulations of the 1:1 EC/DEC electrolyte at 280 and 320 K. The results are summarized in Table S6. The transference coefficients vary little in the temperature interval (within uncertainty). The conductivity increases nearly linearly with the temperature in the temperature interval. The potential contributions due to salt and solvent polarization decrease almost linearly with increasing temperature, so it is appropriate to use the values at 300 K as we have done above. The Seebeck coefficient shows a similar temperature dependence as entropy, which is usually small over such a limited temperature interval. We measure a linear relation between the electric potential difference and temperature in Figure 2d across a wide temperature interval, indicating a small temperature dependence of the Seebeck coefficient. We do not expect the temperature dependence to differ for the electrolyte compositions that we have studied.

## Conclusions

This article presents for the first time a full set of transport coefficients needed to model the concentration and temperature polarization in a lithium-ion battery-relevant electrolyte. The coefficients were determined using a practical procedure recently established to link coefficients under two types of scenarios; for the case that ions are used as electrolyte components and for the case that there are neutral components only. In addition, we report a Seebeck coefficient of 0.86 mV K^–1^ and heats of transfer for the salt varying with concentration from 1.1 to 2.2 kJ mol^–1^, which are small values compared to the Peltier heat. The coefficients allow us to test assumptions that are common in the literature. In the nonisothermal system, all coefficients except the Soret coefficient are significant. The Soret effect can be neglected without a loss of precision in the computation of stationary state polarization under battery operation.

The equilibrium studies of the electrolyte have confirmed earlier results on pair correlation distributions and the electrolyte structure. Diffusion coefficients are supported by less detailed observations in the literature; they have the same order of magnitude. The transport number of the lithium ion in the EC frame of reference is comparable to literature values, about 0.3, but earlier investigations did not include solvent segregation and transport of DEC. Polarization of the salt is the largest contributor to the battery voltage in the stationary state, followed by potential ohmic loss, polarization of component D, and finally thermal polarization. Regarding the alternative cosolvents, we find that DMC produces half the potential loss of DEC, giving in particular a much smaller salt polarization and ohmic loss. Regarding the solvent composition, a higher fraction of component DEC or DMC seems favorable. All terms in eq 1 contributing to the electric potential are relevant and should be taken into account for better battery modeling and understanding. We believe the framework presented here represents an improved starting point for cell-level models (which include porous electrodes and the separator) compared to current state-of-the-art physics-based models.^8,47^

## Experimental Section

### Equilibrium MD Simulations

All MD simulations were performed using the LAMMPS^48^ code. Atomic and ionic interactions were described by the OPLS-AA^49^ potential. The parameters for the solvent molecule atoms were obtained from Ligpargen.^50−52^ The ionic parameters for Li^+^ and PF_6_^–^ ions were taken from Jensen et al.^53^ and Acevedo et al.,^54,55^ respectively. This force field has been thoroughly investigated for modeling of lithium-ion battery electrolytes and is a good compromise of accuracy and computational efficiency. Real-space Lennard-Jones and Coulombic forces were cutoff at 13 Å. A Lennard-Jones tail correction was added to the energy and pressure.^56^ Coulombic forces beyond the cutoff were computed in reciprocal space using a particle–particle particle-mesh solver^57^ with a relative error in forces of 10^–6^. The ionic charges were scaled by a factor of 0.75 to correct for the overestimation of electrostatic interactions between ions in nonpolarizable force fields.^58^ Packmol and Moltemplate were used to prepare initial configurations of the systems by randomly placing solvent molecules Li^+^ and PF_6_^–^ in a simulation box. The 1:1 wt % EC/DEC +1 M LiPF_6_ model electrolyte contained 5520 EC molecules, 4116 DEC molecules, and 920 LiPF_6_. Periodicity was applied in all dimensions.

The equilibration procedure is described in the following. First, the energies of the systems were minimized to avoid particle overlap. Initial equilibration was performed according to the method developed by Molinari et al.^59^ The systems were further equilibrated at a temperature of 350 K or higher and a pressure of 1 atm in the isobaric–isothermal (*NPT*) ensemble using a time step of 1.25 fs in order for the potential energy and density of the systems to stabilize. The temperature and pressure were controlled by the Nosé-Hoover thermostat and barostat^60−62^ using time constants resulting in characteristic fluctuations of 100 and 1000 time steps, respectively. The final equilibration in the *NPT* ensemble was conducted with a temperature of 300 K and pressure of 1 atm while sampling the box volume, and the simulation box size was scaled to the average volume at the end to obtain the correct density. The transport properties were sampled in the canonical ensemble (*NVT*) at 300 K using a time step of 1.25 fs in simulations running for at least 80 ns, which was sufficient to reach the diffusive regime. The Nosé-Hoover thermostat was used in the *NVT* ensemble. The Onsager coefficients of Tables 4 and 6 for the ions and the solvent (mixed component scenario), *L*^++^, *L*^+–^, *L*^––^, *L*^+D^, *L*^–D^, and *L*^DD^, and the RDFs for computing Kirkwood-Buff integrals were obtained using the OCTP module^63^ for LAMMPS. The Onsager coefficients shown in Table 4 and 5 for the salt and the solvents (neutral component scenario), *L*_LL_, *L*_LD_, *L*_DL_, and *L*_DD_, were computed from the set of coefficients in Table 4. The Fickian coefficients in Table 5 were computed using equations in ref^6^. Three parallel simulations were performed from independent starting configurations for the 1:1 EC/DEC +1 M LiPF_6_ system. Data are presented as the mean of three values with the standard deviation. The other systems were simulated only once.

### Nonequilibrium MD Simulations

In order to obtain heats of transfer, a temperature gradient was set up in the *z*-direction by thermostatting the center and edge regions of the simulation box to 280 and 380 K, respectively. Both regions were 4 Å thick, and they were spanning the whole box in the two other dimensions. The edge region was placed such that its center was at the box boundary. The thermostatting was conducted by explicitly rescaling the atom velocities every 10 timesteps. The system was allowed to equilibrate for at least 30 ns using a time step of 1 fs to ensure that a stationary state was reached before sampling the composition profile of the components in the box. The linear momentum of all particles in the box was reset every time step to avoid drift. The volume of the box was held constant during the nonequilibrium simulations. Composition profiles of the components in the simulation box were calculated by sampling the number of the various components in layers of 1 Å thickness. The number of salt molecules inside a layer was defined as the number of cations and anions divided by two.

### Determination of Seebeck Coefficients

#### Cell Assembly

The thermogalvanic cells were assembled as pouch-cells in an argon-filled glovebox. A PC8 pouch-cell laminate from Targray was used as the cell housing. The thermogalvanic cells had a symmetric electrode arrangement, using lithium-chips from Tmax (0.25 mm thick and with a diameter of 15.6 mm). Copper foil was used as a tab for electric potential difference measurements with one part embedded in the cell on the backside of the lithium chips and the other part outside the pouch. A polypropylene tape film was used to reinforce the seal around the tab. A stack of 4 Whatman Glass Microfibre Filters GF/D (no 1823070, pore diameter of 2.7 μm) were used as a separator. The stack was sandwiched between the two electrodes and had a thickness of 1.8 mm after vacuum sealing. The electrolyte was 1 M LiPF_6_ in a 1:1 wt % EC/DEC (LP40) from Gotion. Electrolyte was added to the separators until the separators were soaked but not dripping, approximately 1 mL per cell. The pouch cells were sealed with an Audion VMS 53 Vacuum Chamber. The lowest pressure was reached after 15 s, and the cells spent 25–45 s at this pressure before the cells were sealed. We found no dependence on the time spent under vacuum.

#### Thermogalvanic Cell Measurements

Prior to measurement, the cells were equilibrated by short-circuiting and allowed to reach a stable electric potential difference at isothermal conditions. The thermogalvanic cell was sandwiched between two copper plates within a frame of two aluminum plates (see Figure 2a). A temperature gradient was applied by circulating water in the aluminum frames (see Figure 2a) using two water baths (Grant Ecocool 150R) set to different temperatures. Hot water was circulating in the top plate and cold water in the bottom plate. The electric potential difference between the hot electrode (defined as the positive electrode) and the cool electrode (defined as the negative) was recorded with an Agilent 34970A Data acquisition/Switch unit. A bias potential of typically ±0.3 mV was recorded prior to and in-between the measurements and subtracted from the reading. Type K thermocouples were placed between the copper plates to measure the external temperature difference during the experiment.

The internal temperature difference was found from a calibration experiment with thermocouples embedded in the pouch, cf. ref (39). The temperature between the cell housing and the lithium electrode was measured by two type K thermocouples stripped of the insulation in three Li-symmetric cells. At the same time, the external temperature difference was controlled. The ratio of the two differences was 0.66 ± 0.06.^39^

## Supporting Information Available

Calculation of thermodynamic factors from Kirkwood-Buff integrals; determination of heats of transfer and Seebeck coefficients; and isothermal diffusion coefficients for the additional electrolyte systems (PDF)
