# Supplementary material for: Effect of the Ion, Solvent, and Thermal Interaction Coefficients on Battery Voltage
Source: J Am Chem Soc. 2024 Feb 10;146(7):4592–604. doi: 10.1021/jacs.3c11589 (PMC10885156; doi:10.1021/jacs.3c11589)
Supplement: Supplementary file 1 — ja3c11589_si_001.pdf [file ja3c11589_si_001.pdf]

Supporting Information: The effect of ion-, solvent- and thermal interaction coefficients on battery voltage

Øystein Gullbrekken,† Astrid Fagertun Gunnarshaug,‡ Anders Lervik,‡ Signe Kjelstrup,‡ and Sondre Kvalvåg Schnell*,†

†*Department of Materials Science and Engineering, Norwegian University of Science and Technology, NTNU, N-7491 Trondheim, Norway*

‡*PoreLab, Department of Chemistry, Norwegian University of Science and Technology, NTNU, N-7491 Trondheim, Norway*

E-mail: sondre.k.schnell@ntnu.no

Electrolyte thermodynamic factors

The thermodynamic factor is a way to describe deviations from ideality of a mixture. The relation between the common activity coefficient and the thermodynamic factor is unique, and a transformation from one set of coefficients to another can be done. The thermodynamic factors are defined by,

$$\Gamma_{ij}^{x} = \delta_{ij} + x_i \left(\frac{\partial \ln \gamma_i}{\partial x_j} \right)_{T,p,\sum} \tag{1}$$

where δ_{ij} is the Kronecker delta, γ_i the activity coefficient of component i, and the subscript $\sum$ indicates that the partial derivative is taken at constant mole fraction of all components, except for the nth one. Superscript x indicates that the thermodynamic factors are calculated using mole fractions. Thermodynamic factors can also be calculated using the concentration

as a basis[S1]. Γ_{ij}^{x} is determined from the equilibrium simulations by evaluating the Kirkwood-Buff integrals[S2,S3]:

$$G_{ij} = 4\pi \int_0^\infty \left(g_{ij}(r) - 1\right) r^2 \mathrm{d}r, \tag{2}$$

where $g_{ij}(r)$ is the pair distribution function. The radial distribution functions were calculated using the OCTP plugin[S4] for LAMMPS with the finite-size correction method of van der Vegt et al.[S5,S6]. For evaluating the integrals in Eq. (2), we have used the finite-size correction of Krüger et al.[S7] and one example of the extrapolation is shown in Figure S1 for the 1 M $LiPF_6$ in EC:DEC system.

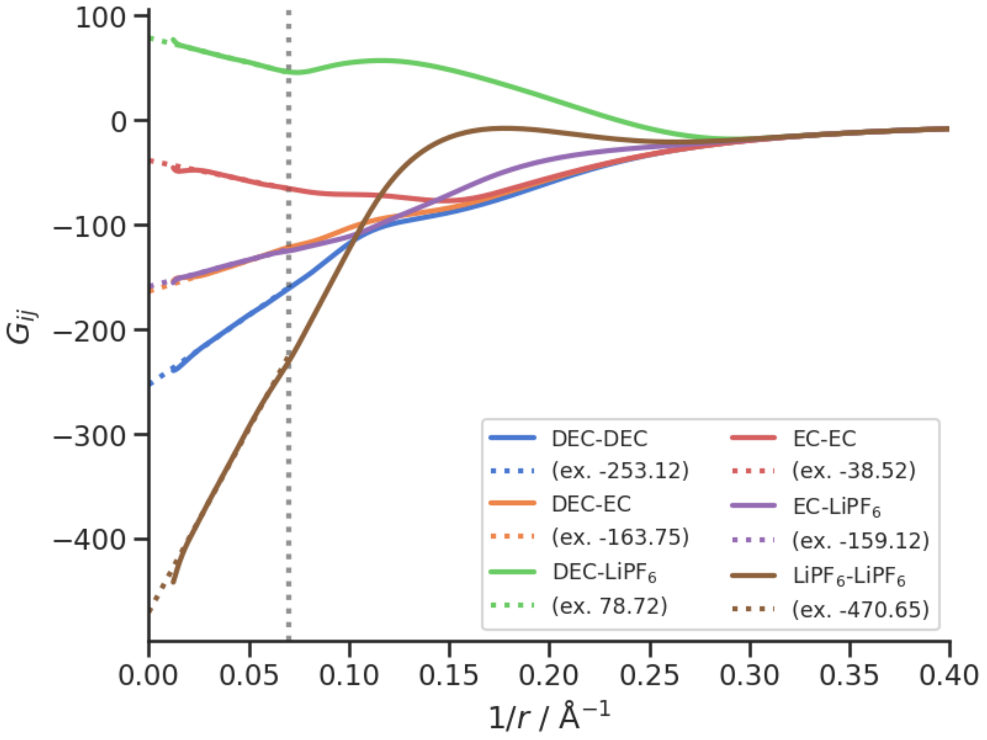

Figure S1: Calculation of G_{ij} by linear extrapolation for the 1 M $LiPF_6$ in EC:DEC system. The dotted vertical line shows the start of the linear extrapolation and the extrapolated values are given in the legend (as the values following “ex.”).

Relations between G_{ij} and Γ_{ij}^{x} for a ternary system are given by Liu et al.[S3] and Krishna

et al.[S8]:

$$
\begin{aligned}
\Gamma_{11}^{x} = -\frac{1}{\eta}\big(& - c_2c_3G_{22} - c_2 + 2c_2c_3G_{23} - c_2c_3G_{33} - c_3 \\
& + c_1\left(c_2G_{12} - c_2G_{22} - 1 + c_2G_{23} - c_2G_{13}\right)\big),
\end{aligned} \tag{3}
$$

$$
\begin{aligned}
\Gamma_{12}^{x} = -\frac{c_1}{\eta}\big(& c_2G_{12} + c_3G_{12} - c_2G_{13} - c_3G_{13} - c_2G_{22} \\
& + c_2G_{23} - c_3G_{23} + c_3G_{33}\big),
\end{aligned} \tag{4}
$$

$$
\begin{aligned}
\Gamma_{21}^{x} = \frac{c_2}{\eta}\big(& c_1G_{11} - c_1G_{12} - c_3G_{12} - c_1G_{13} + c_3G_{13} \\
& + c_1G_{23} + c_3G_{23} - c_3G_{33}\big),
\end{aligned} \tag{5}
$$

$$
\begin{aligned}
\Gamma_{22}^{x} = \frac{1}{\eta}\big(& c_1c_3G_{11} + c_1 - 2c_1c_3G_{13} + c_1c_3G_{33} + c_3 \\
& + c_2\left(c_1G_{11} - c_1G_{12} - c_1G_{13} + 1 + c_1G_{23}\right)\big),
\end{aligned} \tag{6}
$$

where c_i is the molar concentration of i and,

$$
\begin{aligned}
\eta = & c_1 + c_2 + c_3 + c_1c_2\Delta G_{12} + c_2c_3\Delta G_{23} + c_1c_3\Delta G_{13} \\
& - \frac{1}{4}c_1c_2c_3\big(\Delta G_{12}^2 + \Delta G_{23}^2 + \Delta G_{13}^2 - 2\Delta G_{13}\Delta G_{23} \\
& - 2\Delta G_{12}\Delta G_{13} - 2\Delta G_{12}\Delta G_{23}\big),
\end{aligned} \tag{7}
$$

and,

$$
\Delta G_{ij} = G_{ii} + G_{jj} - 2G_{ij}. \tag{8}
$$

For the evaluation of the thermodynamic factors, EC has been taken as the reference (component 3).

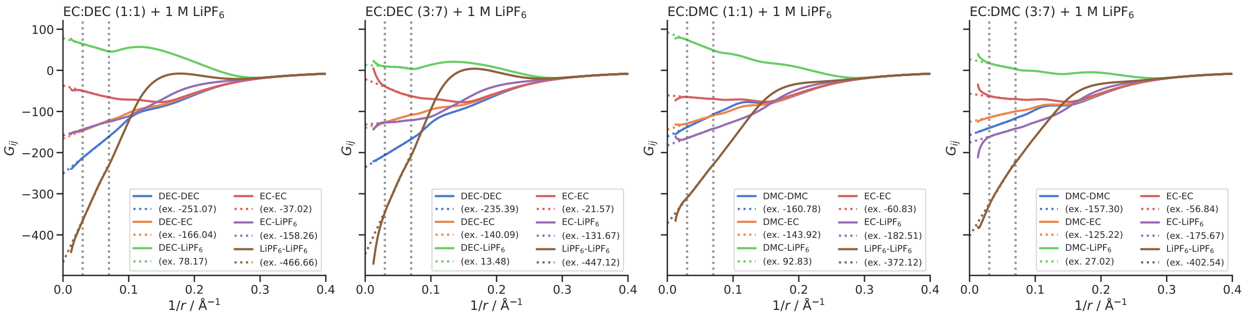

Figure S2: Kirkwood-Buff integrals as a function of inverse distance for the four electrolytes studied. The regions used for linear regression to find the intercepts of the curves are displayed by dotted vertical lines.

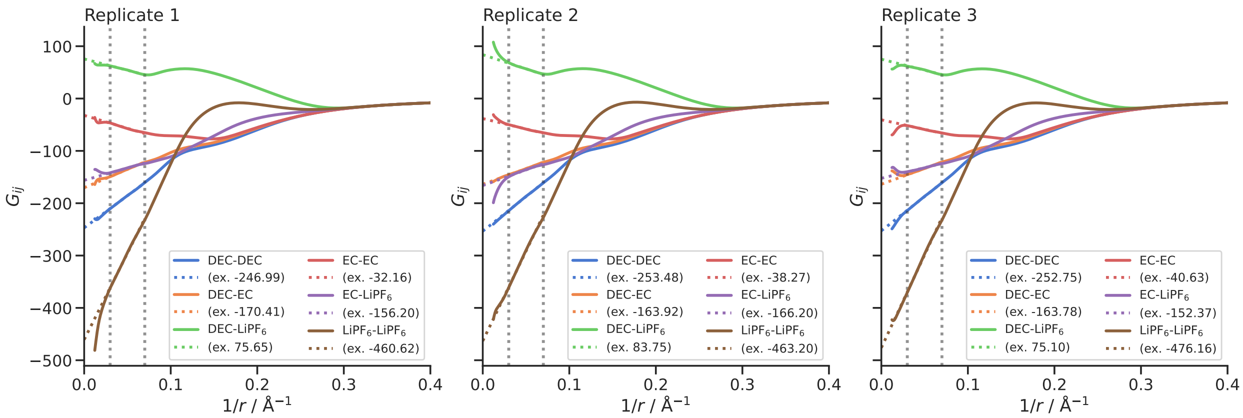

Figure S3: Kirkwood-Buff integrals as a function of inverse distance for the three replicates of the 1:1 EC:DEC electrolyte. The regions used for linear regression to find the intercepts of the curves are displayed by dotted vertical lines.

Thermal coefficients

The heat of transfer can be computed from composition gradients once we have the thermodynamic factors, Γ^x_{ij}. In the stationary state for transport of salt, we obtain

$$q^*_{\mathrm{L}} = \left(\frac{J'^N_q}{J_{\mathrm{L}}}\right)_{J_{\mathrm{D}}=0,j=0,dT=0} = -T\left(\frac{\nabla\mu_{\mathrm{L},T}}{\nabla T}\right)_{J_{\mathrm{L}}=J_{\mathrm{D}}=0,j=0} \tag{9}$$

An equivalent expression can be found for D. In both cases, ∇T is directly measured and $\nabla \mu_{\mathrm{i},T}$ for $i = \mathrm{L}$ or D is determined from

$$\nabla \mu_{i,T} = \sum_{j=1}^{2} \Gamma_{ij}^{x} \frac{RT}{x_j} \nabla x_j, \tag{10}$$

where R is the gas constant and x_j is the mole fraction of (independent) components $j = \mathrm{L}$ or D. The gradient in the mole fraction ∇x_j is determined in non-equilibrium simulations, and Γ_{ij}^{x} is determined from equilibrium simulations by evaluating equation (2).

The aim of the present investigation is to compute the electric potential gradient across the electrolyte from the last line in equation 2 in the main text. This can be done with knowledge of the conductivity $L_{\varphi\varphi}/T$ and the transference coefficients $t_i = F(L_{i\varphi}/L_{\varphi\varphi})$. These coefficients can be obtained from equilibrium simulations, using fluctuation-dissipation theorems. The heat of transfer can be determined from non-equilibrium simulations by setting up a heat flux and measuring the resulting composition gradients.

The Peltier coefficient on the other hand is not directly obtainable from simulations. It will here be determined from Seebeck coefficient measurements. By using the Onsager relations, we obtain the identity

$$\left(\frac{\pi}{T}\right)_{dT=0,d\mu_i=0} = -F\left(\frac{\Delta\varphi}{\Delta T}\right)_{j=0,d\mu_i=0}. \tag{11}$$

The expression applies to a subsystem as well as to the whole measuring cell. The single contributions to the Peltier heat was obtained for this cell from the entropy balance[S9]:

$$\pi = T\left(S_{\mathrm{Li}^+}^{*} + S_{\mathrm{e}^-}^{*} - S_{\mathrm{Li}}\right) + \sum_{i=1}^{2} t_i q_i^{*} \tag{12}$$

Here S_i^* are transported entropies of the lithium ion and the electron, respectively, S_{Li} is the entropy of lithium. The heat of transfer and the transference coefficients were defined above. The transported entropy of the electron is assumed to be small.

Table S1: Thermodynamic factors (Γ_{ij}^x, L = $LiPF_6$, D = DEC or DMC) used to calculate heats of transfer.

System	Γ_{LL}^x	Γ_{LD}^x	Γ_{DL}^x	Γ_{DD}^x
1:1 EC:DEC + 1 M $LiPF_6$	1.45	−0.28	−0.94	1.19
3:7 EC:DEC + 1 M $LiPF_6$	1.41	−0.22	−0.72	1.20
1:1 EC:DMC + 1 M $LiPF_6$	1.40	−0.26	−1.00	1.16
3:7 EC:DMC + 1 M $LiPF_6$	1.35	−0.21	−0.75	1.16

The equilibrium structure of the electrolyte

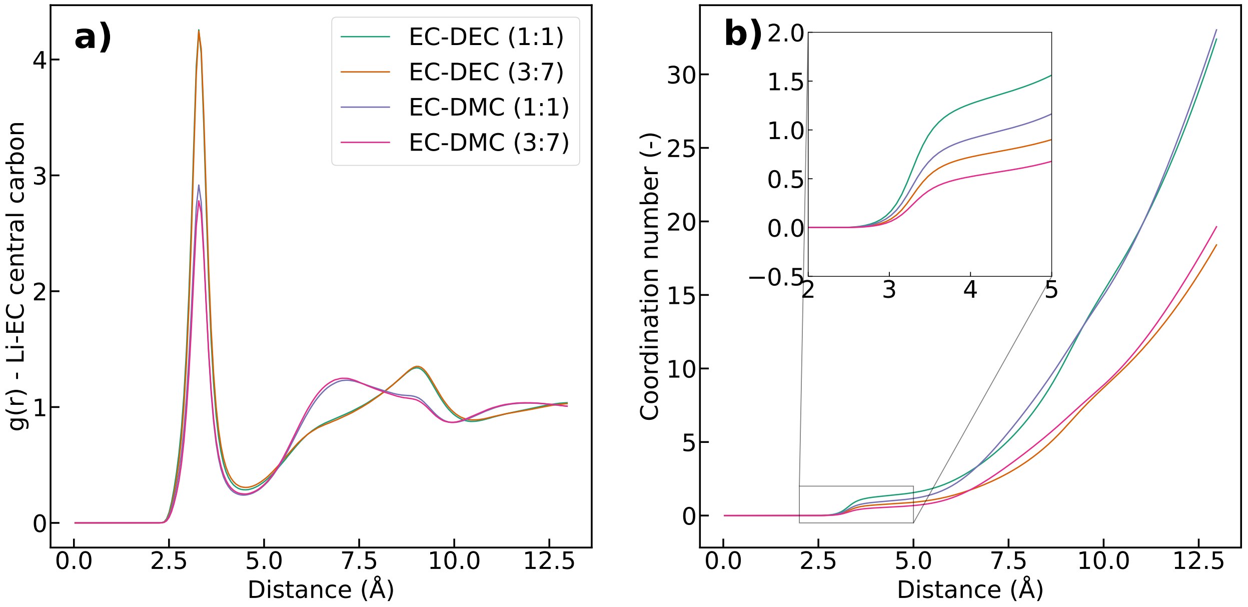

Figure S4: (a) Radial distribution functions and (b) coordination numbers of the central carbon atom of EC around Li.

In Tables S2, S3 and S4, the coefficients are obtained using Eq. (13) in the barycentric (B) frame of reference, and converted to the EC- and DEC frames of reference. The conversion is shown in Ref. S1. As described by Liu et al.[S3], the barycentric Onsager coefficients, Λ_{ij}, can be directly obtained in MD simulations from the particle displacements as a function of

time,

$$\begin{aligned}\Lambda_{ij} =& \frac{1}{6}\lim_{m\to\infty}\frac{1}{N}\frac{1}{m\Delta t}\langle(\sum_{l=1}^{N_i}(r_{l,i}(t+m\Delta t)-r_{l,i}(t)))\\ &\times(\sum_{k=1}^{N_j}(r_{k,j}(t+m\Delta t)-r_{k,j}(t)))\rangle,\end{aligned} \tag{13}$$

where Δt is the simulation time step, N the total number of molecules, and $r_{l,k}(t)$ the position of molecule/particle l of species k at time t.

Table S2: Diffusion coefficients for the mixed component scenario of the isothermal electrolyte of 1:1 wt.% EC:DEC + 1 M $LiPF_6$ using the barycentric (B), EC- and DEC frames of reference.

Frame of reference	B	EC	DEC
Coefficient	Value $\times$ 10^{-11} m^2/s	Value $\times$ 10^{-11} m^2/s	Value $\times$ 10^{-11} m^2/s
L^{++}	0.35 ± 0.04	0.8 ± 0.1	0.3 ± 0.1
L^{--}	0.7 ± 0.2	1.3 ± 0.2	1.0 ± 0.2
L^{+-}	0.08 ± 0.02	0.56 ± 0.05	0.2 ± 0.1
L^{D+}	0.5 ± 0.1	2.4 ± 0.2	
L^{D-}	-0.3 ± 0.1	1.8 ± 0.2	
L^{DD}	2.8 ± 0.2	11.3 ± 1.3	
L^{E+}	-0.9 ± 0.1		0.1 ± 0.3
L^{E-}	-1.0 ± 0.1		0.9 ± 0.1
L^{EE}	6.1 ± 0.9		20.3 ± 2.3
L^{ED}	-3.4 ± 0.4		

When formulated with fluxes and forces, as defined by the entropy production, eq. 1 in the main text, the fluctuation dissipation theorem refers to coefficients of the flux-force matrix, eq. 2 in the main text. The coefficients dimension in eq. 2 follow, also for isothermal systems, when the factor $1/T$ is contained in the force.

Table S3: Diffusion coefficients for the mixed component scenario of the isothermal electrolyte of 3:7 wt.% EC:DEC + 1 M $LiPF_6$ using the EC frame of reference. Transference coefficients, t, and transport numbers, τ, are dimensionless.

Coefficient	Value ($\times 10^{-11}$ m^2/s)	Value ($\times 10^{-9}$ K mol^2/(J m s))
L^{++}	1.1	14.9
L^{--}	1.6	21.4
L^{+-}	0.9	12.3
L^{D+}	4.4	60.0
L^{D-}	4.1	55.9
L^{DD}	24.9	341.9
κ	0.25 S/m	
t_L	−1.04	
t_D	0.47	
τ_+	0.22	

Table S4: Diffusion coefficients for the mixed component scenario of the isothermal electrolyte of 3:7 wt.% EC:DMC + 1 M $LiPF_6$ using the EC frame of reference. Transference coefficients, t, and transport numbers, τ, are dimensionless.

Coefficient	Value ($\times 10^{-11}$ m^2/s)	Value ($\times 10^{-9}$ K mol^2/(J m s))
L^{++}	1.8	31.1
L^{--}	2.3	38.7
L^{+-}	1.3	22.1
L^{D+}	9.5	159.1
L^{D-}	8.0	134.4
L^{DD}	71.8	1205.8
κ	0.54 S/m	
t_L	−0.87	
t_D	1.29	
τ_+	0.35	

Table S5: Potential contributions to cell voltage in the isothermal case

Electrolyte	A (10^{-11} mol^2/(J m s))	B	$1/\kappa$ (Ω m)	$\frac{t_D}{F^2}\frac{B}{A}$ (Ω m)	$\frac{t_L}{F^2}\left(-\frac{B}{A}\frac{l_{DD}}{l_{DL}}+t_D\frac{T}{l_{DL}}\right)$ (Ω m)
1:1 EC:DEC	-6.3 ± 2.6	-1.3 ± 0.1	4.4 ± 0.7	2.4 ± 1.7	10.2 ± 4.6
3:7 EC:DEC	−7.8	−1.2	4.0	0.8	9.3
1:1 EC:DMC	−14.7	−1.2	2.2	0.7	5.3
3:7 EC:DMC	−23.0	−1.1	1.9	0.7	3.3

Table S6: Diffusion coefficients for the mixed component scenario of the isothermal electrolyte of 1:1 wt.% EC:DEC + 1 M $LiPF_6$ using the EC frame of reference at 280 and 320 K. Transference coefficients, t, and transport numbers, τ, are dimensionless. Potential contributions to cell voltage in the bottom section. Mean values and standard deviations from two replicas.

Temperature	280 K	320 K
Coefficient	Value ($\times$ 10^{-11} m^2/s)	Value ($\times$ 10^{-11} m^2/s)
L^{++}	0.4	1.8
L^{--}	0.5 ± 0.1	2.5
L^{+-}	0.2	1.2
$L^{\mathrm{D}+}$	1.0 ± 0.3	5.3 ± 0.4
$L^{\mathrm{D}-}$	0.6	3.9 ± 0.2
L^{DD}	5.1 ± 1.6	25.2 ± 1.0
κ	(0.14 ± 0.04) S/m	(0.50 ± 0.01) S/m
t_{L}	-0.90 ± 0.12	-0.92
t_{D}	1.31 ± 1.11	0.88 ± 0.13
τ_+	0.33 ± 0.09	0.31 ± 0.01
Potential contributions to cell voltage		
$1/\kappa$ (Ω m)	7.8 ± 2.4	2.0
$\frac{t_{\mathrm{D}}}{F^2}\frac{B}{A}$ (Ω m)	5.8 ± 6.1	0.8 ± 0.2
$\frac{t_{\mathrm{L}}}{F^2}\left(-\frac{B}{A}\frac{l_{\mathrm{DD}}}{l_{\mathrm{DL}}} + t_{\mathrm{D}}\frac{T}{l_{\mathrm{DL}}}\right)$ (Ω m)	15.5 ± 3.5	3.9 ± 0.5

References

(S1) Kjelstrup, S.; Gunnarshaug, A. F.; Gullbrekken, Ø.; Schnell, S. K.; Lervik, A. Transport coefficients for ion and solvent coupling. The case of the lithium-ion battery electrolyte. *The Journal of Chemical Physics* **2023**, *159*, 034104.

(S2) Kirkwood, J. G.; Buff, F. P. The Statistical Mechanical Theory of Solutions. I. *The Journal of Chemical Physics* **1951**, *19*, 774–777.

(S3) Liu, X.; Martín-Calvo, A.; McGarrity, E.; Schnell, S. K.; Calero, S.; Simon, J.-M.; Bedeaux, D.; Kjelstrup, S.; Bardow, A.; Vlugt, T. J. H. Fick Diffusion Coefficients in Ternary Liquid Systems from Equilibrium Molecular Dynamics Simulations. *Industrial & Engineering Chemistry Research* **2012**, *51*, 10247–10258.

(S4) Jamali, S. H.; Wolff, L.; Becker, T. M.; de Groen, M.; Ramdin, M.; Hartkamp, R.; Bardow, A.; Vlugt, T. J. H.; Moultos, O. A. OCTP: A Tool for On-the-Fly Calculation of Transport Properties of Fluids with the Order-n Algorithm in LAMMPS. *Journal of Chemical Information and Modeling* **2019**, *59*, 1290–1294.

(S5) Ganguly, P.; van der Vegt, N. F. A. Convergence of Sampling Kirkwood–Buff Integrals of Aqueous Solutions with Molecular Dynamics Simulations. *Journal of Chemical Theory and Computation* **2013**, *9*, 1347–1355, PMID: 26587597.

(S6) Milzetti, J.; Nayar, D.; van der Vegt, N. F. A. Convergence of Kirkwood–Buff Integrals of Ideal and Nonideal Aqueous Solutions Using Molecular Dynamics Simulations. *The Journal of Physical Chemistry B* **2018**, *122*, 5515–5526, PMID: 29342355.

(S7) Krüger, P.; Schnell, S. K.; Bedeaux, D.; Kjelstrup, S.; Vlugt, T. J. H.; Simon, J.-M. Kirkwood–Buff Integrals for Finite Volumes. *The Journal of Physical Chemistry Letters* **2013**, *4*, 235–238, PMID: 26283427.

(S8) Krishna, R.; van Baten, J. M. The Darken Relation for Multicomponent Diffusion in Liquid Mixtures of Linear Alkanes: An Investigation Using Molecular Dynamics (MD) Simulations. *Industrial & Engineering Chemistry Research* **2005**, *44*, 6939–6947.

(S9) Spitthoff, L.; Gunnarshaug, A. F.; Bedeaux, D.; Burheim, O.; Kjelstrup, S. Peltier effects in lithium-ion battery modeling. *The Journal of Chemical Physics* **2021**, *154*, 114705.
